# Professionalism and Ethics in Medical and Dental Education: A Survey of Student Perceptions and Experiences

**DOI:** 10.7759/cureus.76113

**Published:** 2024-12-21

**Authors:** Sana Noor, Anusha Nauroz Ali, Azhar Ejaz, Aafia Malik, Khadijah Nadeem, Zain Fatima, Aatika Shakoor, Amina Khalil

**Affiliations:** 1 Community Medicine, Avicenna Medical and Dental College and Hospital, Lahore, PAK; 2 Medicine and Surgery, Avicenna Medical and Dental College and Hospital, Lahore, PAK; 3 General Surgery, Worcestershire Acute Hospitals NHS Trust, Worcester, GBR; 4 Surgical Unit 1, Services Hospital, Lahore, PAK; 5 Psychiatry, Herefordshire and Worcestershire Health and Care NHS Trust, Worcester, GBR; 6 Psychiatry, Jinnah Hospital, Lahore, PAK; 7 General Medicine, Avicenna Medical and Dental College and Hospital, Lahore, PAK; 8 General Surgery, Nishtar Hospital, Multan, PAK

**Keywords:** cross-sectional study, gender differences, healthcare education, medical ethics, professionalism, unprofessional behavior

## Abstract

Background: Professionalism and ethical behavior are critical components of medical practice, yet gaps in ethical education among medical students remain a concern. This study addresses the need to assess perceptions and experiences regarding professionalism among medical students.

Objective: This study aims to evaluate medical students' awareness, perceptions, and experiences related to professionalism and ethical practices.

Methodology: A cross-sectional study was conducted using stratified random sampling across seven medical institutions (both public and private). A structured, self-administered questionnaire was distributed to 815 Bachelor of Medicine and Bachelor of Surgery (MBBS) and Bachelor of Dental Surgery (BDS) students, yielding a response rate of approximately 90%. Data analysis involved IBM SPSS Statistics for Windows, Version 27 (Released 2020; IBM Corp., Armonk, New York, United States), using descriptive statistics, Mann-Whitney U, and Chi-square tests.

Results: The sample comprised predominantly young adults (67.4% aged 20-22), with more females (64%) than males (36%). While 94% of participants were familiar with professional ethics, only 40% had completed a formal course in medical ethics, and 26.1% had received additional professional ethics training. A majority (59.3%) engaged in self-directed study on professionalism. Regarding unprofessional behavior, 61% observed a lack of medical dignity, 48.5% witnessed disrespect for cultural differences, and 48.5% noticed the failure to introduce oneself. Other observed unprofessional behaviors included performing procedures without supervision (50.3%) and unprofessional behavior in hospital corridors (46.5%). Eating or drinking in hospital hallways was observed by 43.8% of participants. These findings reveal a high awareness of professionalism and medical ethics, although unprofessional behavior remains common in healthcare settings. Gender differences were significant for training and self-directed ethics study (p < 0.05). The instrument used for the study demonstrated good reliability (Cronbach’s alpha = 0.82).

Conclusion: There is a critical need for integrating comprehensive ethics training into medical education to address observed gaps and improve professional conduct among future healthcare providers. The findings underscore the urgent need for integrating comprehensive ethics training into medical education. Addressing observed gaps in ethical understanding and behavior could inform curriculum development and foster professionalism among future healthcare providers. Such enhancements may ultimately improve the quality of patient care and strengthen public trust in the healthcare system.

## Introduction

Professionalism and ethical behavior are foundational to medical and dental practice, shaping patient care and team dynamics within healthcare settings [1]. Early exposure to ethical dilemmas during medical and dental education plays a pivotal role in developing students’ ethical frameworks and professional behavior. As future healthcare professionals, students are expected to uphold high ethical standards, making it crucial to understand their perceptions of and responses to unprofessional behavior in order to enhance healthcare education [2].

Mak-van der Vossen et al. [3] found that students frequently witness or engage in unprofessional actions, such as disrespect toward patients and peers, which can erode their professional identity. Similarly, Pavithra et al. [4] highlighted issues like breaches of patient confidentiality, poor communication, and insensitivity to cultural differences in clinical settings. Educational culture and individual values influence students’ perceptions, with early-year students often perceiving unprofessional conduct as less concerning, while senior students develop a more nuanced understanding of ethical standards [5,6]. Research has emphasized the prevalence of unprofessional behavior in medical education [3,7].

Studies suggest that frequent exposure to unethical behaviors can lead students to view them as normal. For example, Jamalabadi et al. [8] observed that students may dismiss minor professionalism violations, such as tardiness or inappropriate comments, especially when exhibited by senior staff. This normalization risks creating an environment where unethical practices are implicitly accepted. On the other hand, Buhumaid et al. [9] demonstrated that interventions integrated early into the curricula can effectively address and reduce unprofessional behavior.

Güner et al. [10] further noted that students’ attitudes toward unethical behavior differ based on their year of study and clinical exposure. Younger students may perceive certain behaviors as part of the clinical culture, while senior students, informed by more experience, adopt a more critical stance. This underscores the importance of continuous ethics education to promote professionalism throughout medical and dental training. In Pakistan, the normalization of unprofessional behavior in busy teaching hospitals highlights the pressing need for robust ethical guidelines and comprehensive ethics training. Given the evolving landscape of medical education in Pakistan, this study seeks to assess students’ perceptions of and involvement in unprofessional behaviors, with the aim of identifying areas for targeted interventions. The primary objective is to explore how medical and dental students perceive unprofessional behaviors in clinical practice, while the secondary goal is to evaluate the acceptability of these actions.

## Materials and methods

Study design and setting

This cross-sectional study was conducted to assess the perceptions and experiences of medical students regarding professionalism and ethical practices. Data were collected from both public and private medical institutions, including private colleges (Avicenna Medical College, Rashid Latif Medical College, Amna Inayat Medical College, Nawaz Sharif Medical College) and public institutions (Quaid-e-Azam Medical College, Gujranwala Medical College, Khyber Medical College).

Inclusion and exclusion criteria

Students enrolled in Bachelor of Medicine and Bachelor of Surgery (MBBS) and Bachelor of Dental Surgery (BDS) programs across all years of study (first to final year) were included to capture a wide range of perspectives. Participants had to be currently enrolled and willing to complete the questionnaire. Exclusion criteria included incomplete responses or refusal to provide informed consent. These criteria were designed to ensure that data reflected the active student population with adequate exposure to clinical and academic environments.

Sample and data collection

A stratified random sampling technique was used to ensure adequate representation from different institutions and years of study. The student population was divided into strata based on the type of degree (MBBS and BDS) and the year of study (first to final year). Each stratum was proportionally represented, and efforts were made to recruit a diverse range of participants from each year and institution. A structured, self-administered questionnaire (Appendix), adapted from Jamalabadi and Ebrahimi [8], was used to collect data. The questionnaire was designed to assess participants' demographics, awareness of professional ethics, prior exposure to ethics training, and their observations of unprofessional behaviors.

Reliability and validity

The questionnaire underwent rigorous validation to ensure reliability and accuracy. Face and content validity were established by consulting four professors, four PhD students in medical ethics, and 15 clinical students. Iterative revisions were made based on their feedback. To assess reliability, a pilot test was conducted with 30 medical students. The questionnaire was readministered to the same group after two weeks to measure test-retest reliability, which was found to be 0.80. Internal reliability, measured using Cronbach's alpha, was 0.79, indicating acceptable reliability levels.

Statistical analysis

Data were analyzed using IBM SPSS Statistics for Windows, Version 27 (Released 2020; IBM Corp., Armonk, New York, United States) under the guidance of a biostatistician to ensure the appropriate application of statistical methods. Descriptive statistics, including frequencies and percentages, summarized demographic characteristics, familiarity with ethics, and experiences with unprofessional behaviors. Measures of central tendency (mean, median) and dispersion (standard deviation) were computed to assess data distribution. Normality was checked using skewness and kurtosis, and non-parametric tests were applied due to non-normal distributions.

Mann-Whitney U tests compared independent groups (e.g., gender, degree type) on variables related to ethical training and professional behaviors, while Chi-square tests explored associations between categorical variables such as completion of ethics courses and engagement in ethics training. Adjustments for confounding factors (e.g., prior ethics training, clinical exposure, institutional differences) were made using stratified analyses to isolate the effects of key variables. Cross-tabulations provided additional insights into relationships between demographic variables and perceptions of professional behaviors.

Minimizing bias

To minimize self-reporting bias, participants were assured of confidentiality and encouraged to provide honest responses without fear of repercussions. The stratified sampling approach reduced selection bias, and iterative revisions to the questionnaire following pilot testing ensured clarity and minimized biases.

Ethical considerations

This study adhered to the ethical standards of the Helsinki Declaration. Ethical approval was granted by the Institutional Review Board (IRB) of the Avicenna Research Ethics Committee (IRB-46/11/23/AVC, dated November 22, 2023). Informed consent was obtained from the participants, who were assured of anonymity and confidentiality. Participation was voluntary, and students could withdraw at any time without repercussions.

## Results

The majority of participants fall into the 20-22 years age range, with 549 individuals (67.4%) from this group. A smaller portion, 163 participants (20%), is in the 23-25 years range, while 83 participants (10.2%) are in the 17-19 years category. Only 10 participants (1.2%) fall within the 20-22 years age group, and nine participants (1.1%) belong to the combined age group of 20-22 years and 23-25 years. This distribution shows that most participants are young adults, predominantly in the 20-22 years range.

There is a noticeable gender imbalance in the sample, with 522 female participants (64%) and 293 male participants (36%). This indicates that a larger proportion of the sample consists of female participants compared to male participants.

Among the participants, 674 are MBBS students (82.7%), while 141 are BDS students (17.3%). This data reveals a significant skew toward MBBS students, who make up the majority of the sample, with a much smaller group of BDS students.

**Figure 1:**
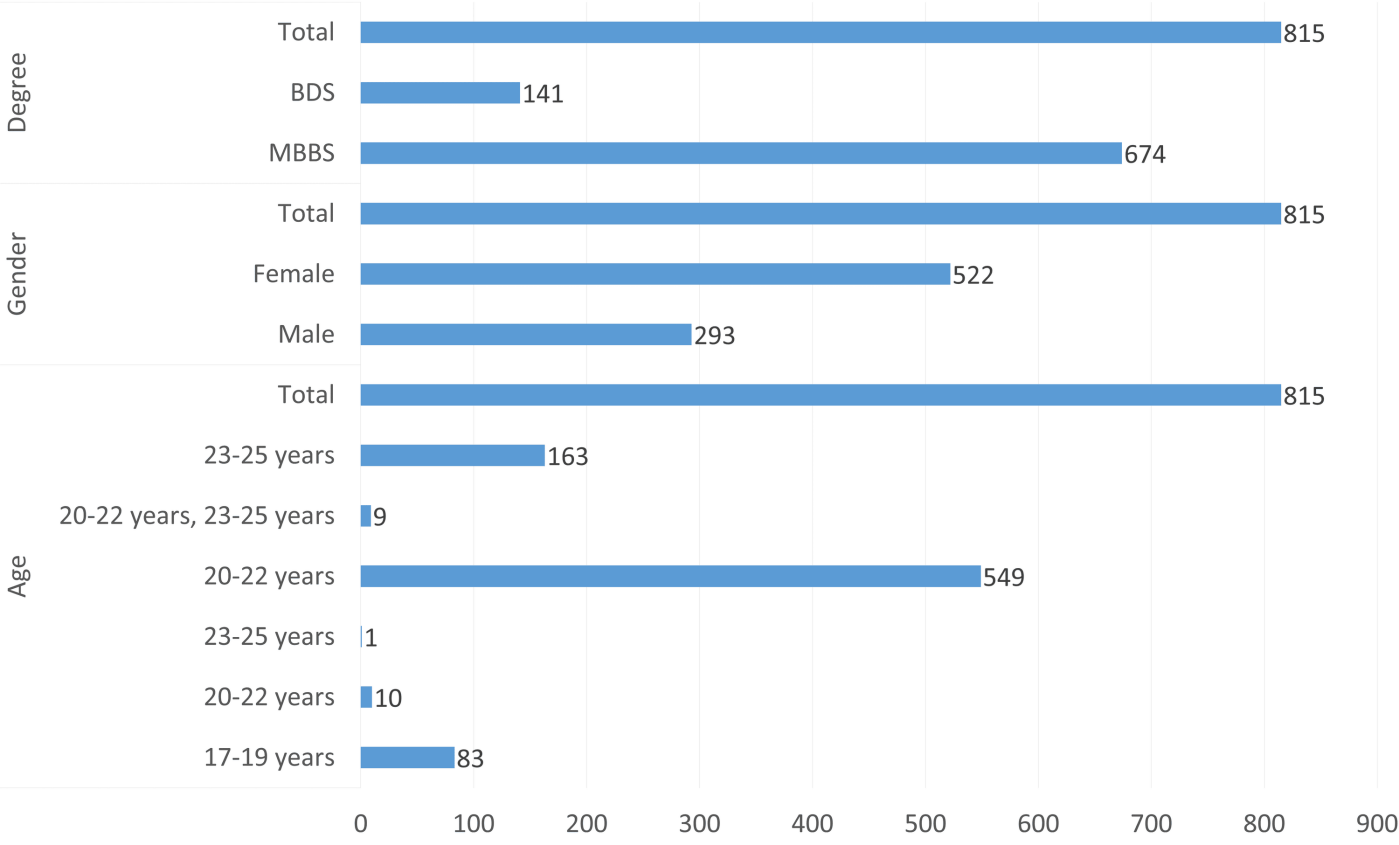
Participants' demographic data

The participants are relatively well-distributed across different years of study. There are 80 first-year students (9.8%), 225 second-year students (27.6%), 217 third-year students (26.6%), 198 fourth-year students (24.3%), and 86 fifth-year students (10.6%). However, nine participants (1.1%) had missing data. This shows that second- and third-year students make up the largest groups, followed by fourth-year students, as shown in Figure 1.

A vast majority of participants, 766 individuals (94%), are familiar with the meaning and application of professional ethics, while only 49 participants (6%) are not. This indicates a high level of awareness and understanding of professional ethics among the sample.

When asked about prior exposure to medical ethics courses, 326 participants (40%) reported having taken a course in medical ethics, while 489 participants (60%) had not. This suggests that a significant portion of the sample has not received formal education in medical ethics, even though it is a crucial area for their professional development.

Regarding other professional ethics training, 213 participants (26.1%) had completed additional training beyond medical ethics courses, while 602 participants (73.9%) had not. This further indicates a need for more comprehensive training in professional ethics outside of formal courses to better prepare future healthcare professionals.

In terms of self-directed learning, 483 participants (59.3%) have engaged in an independent study focused on professionalism, while 332 participants (40.7%) have not. This shows a strong inclination among most participants to pursue their own education on professionalism, even without formal instruction.

Regarding the lack of maintaining medical dignity, 199 participants (24.4%) reported neither observing nor participating in such behavior. The majority, 497 participants (61%), observed a lack of medical dignity in relationships, while 71 participants (8.7%) both observed and participated. Only 48 participants (5.9%) participated without observing. This suggests that while most participants have seen instances of disrespect in medical settings, a smaller group has directly been involved in these situations.

On the issue of respecting religious and cultural differences, 325 participants (39.9%) neither observed nor participated in disrespectful behavior. A larger group, 395 participants (48.5%), reported observing such behavior. Additionally, 51 participants (6.3%) observed and participated, while 44 participants (5.4%) participated without observing. These findings show that while many have witnessed such behavior, only a smaller proportion has actively participated in it.

Regarding the failure to introduce oneself, 395 participants (48.5%) neither observed nor participated, while 301 participants (36.9%) reported having observed this failure to introduce. Additionally, 70 participants (8.6%) both observed and participated, and 49 participants (6%) participated without observing. This indicates that nearly half of the participants have not been involved in this behavior, but a significant portion has witnessed it.

In terms of performing procedures without proper supervision, 410 participants (50.3%) neither observed nor participated. A smaller group, 304 participants (37.3%), observed such occurrences, while 58 participants (7.1%) both observed and participated. Only 43 participants (5.3%) participated without observing. This suggests that half of the participants have not witnessed or engaged in this behavior, while others have observed or participated in it.

Concerning unprofessional behavior in hospital corridors, 296 participants (36.3%) neither observed nor participated in such conversations or mockery. A larger group, 379 participants (46.5%), observed this behavior. Moreover, 97 participants (11.9%) both observed and participated, while 43 participants (5.3%) participated without observing. This shows that more than half of the participants have either observed or participated in such unprofessional behaviors.

**Figure 2:**
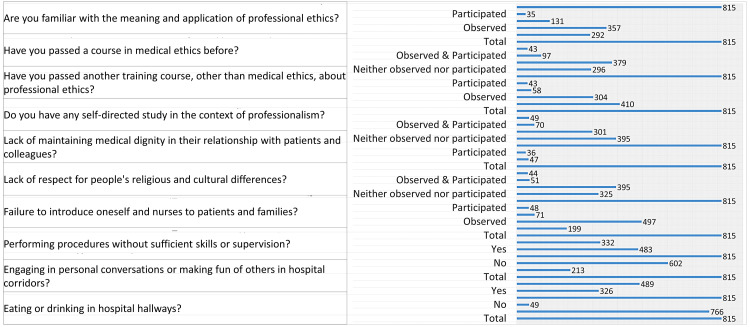
Professional ethics awareness data of participants

When asked about eating or drinking in hospital hallways, 292 participants (35.8%) neither observed nor participated in this behavior. A total of 357 participants (43.8%) observed it, while 131 participants (16.1%) both observed and participated, and 35 participants (4.3%) participated without observing. The data indicates that eating or drinking in hospital hallways is a common occurrence, with many participants having observed it and some having participated as well. These results showed that most participants are aware of and have engaged in various aspects of medical professionalism, with a majority also actively participating in self-directed studies or additional training on the subject (Figure 2). 

**Figure 3:**
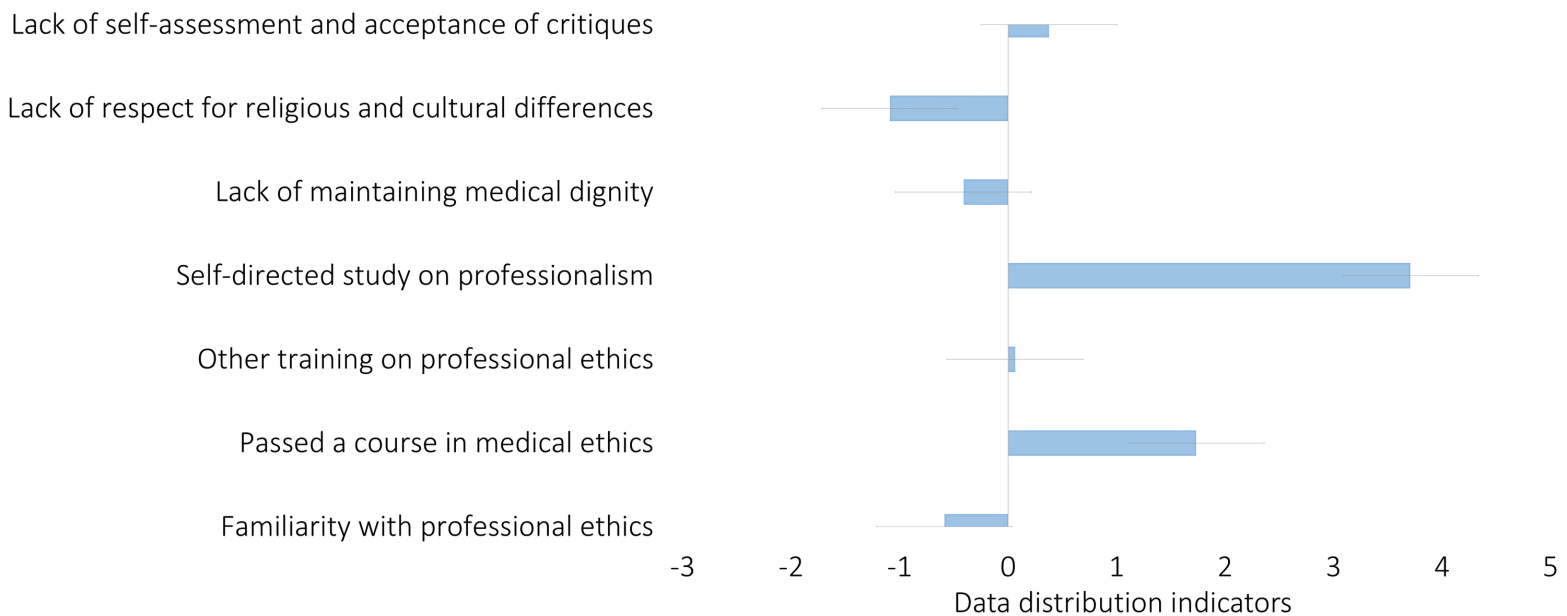
Skewness of professionalism-related variables: distribution analysis of responses on medical ethics and professional behavior indicators

A skewness of 0 suggests a perfectly symmetrical distribution, while positive skewness indicates a longer tail on the right, and negative skewness, a longer tail on the left. In this analysis, variables like "Passed a course in medical ethics" (1.732) and "Self-directed study on professionalism" (3.708) show a strong right skew, meaning many respondents rated these aspects lower, implying a tendency toward less professional behavior (Figure 3). On the other hand, "Lack of maintaining medical dignity" (-0.409) and "Lack of self-assessment" (-0.587) exhibit slight negative skewness, indicating more positive ratings and a perception of fewer unprofessional behaviors. The variable "Other training on professional ethics" (0.065) has a nearly symmetrical distribution, suggesting a balanced response.

The Mann-Whitney U test results for gender differences in various professional ethics-related variables show statistically significant differences across several aspects (Table 1). For "Degree" (U = 68449, p = 0), "Year of study" (U = 66272, p = 0.007), and "Have you passed other training courses on professional ethics?" (U = 61630, p = 0), gender differences were highly significant. Additionally, significant differences were found for "Are you familiar with the meaning and application of professional ethics?" (U = 73056.5, p = 0.01), "Have you passed a course in medical ethics before?" (U = 69219.5, p = 0.008), and "Do you have any self-directed study in the context of professionalism?" (U = 70622.5, p = 0.033). These results suggest that gender plays a role in how participants respond to various professionalism-related questions, with males and females differing significantly in their experiences and perceptions.

**Table 1:** Mann-Whitney U test results with adjustments for confounding factors in ethical perceptions and training MBBS: Bachelor of Medicine and Bachelor of Surgery; BDS: Bachelor of Dental Surgery; OR: odds ratio; CI: confidence interval Adjusted for gender, year of study, prior ethics training, clinical exposure, and institution type

Variable	Mann-Whitney U	Wilcoxon W	Z	Asymp. sig. (2-tailed)	Adjusted OR/β (95% CI)	Adjusted for confounders
Degree (MBBS vs. BDS)	68449	111520	-3.798	0	1.35 (1.10-1.65)	Gender, prior ethics training, institution type
Year of study (first–final)	66272	107888	-2.707	0.007	1.20 (1.05-1.40)	Gender, institution type
Familiarity with professional ethics	73056.5	209559.5	-2.573	0.01	1.25 (1.05-1.50)	Gender, prior training, self-directed study
Passed a course in medical ethics	69219.5	112290.5	-2.651	0.008	1.50 (1.15-1.95)	Gender, clinical exposure, institution type
Other training in professional ethics	61630	104701	-6.048	0	1.75 (1.45-2.10)	Gender, year of study, prior training
Self-directed study on professionalism	70622.5	113693.5	-2.132	0.033	1.10 (1.00-1.30)	Clinical exposure, institution type

Table 2 provides the statistical results of various variables related to professional ethics. Most of the variables related to ethics, including "Lack of maintaining medical dignity," "Lack of respect for cultural differences," and "Failure to comply with regulations," show highly significant results with a p-value of 0 (asymptotic significance), indicating that there is a strong association between these behaviors and the responses from the study participants. These findings suggest that unprofessional behavior in these areas is perceived as a prevalent issue among the sample population, with respondents agreeing that such behaviors are widespread or impactful in their respective fields. The Chi-square tests for these categories reveal that the observed frequencies significantly deviate from expected frequencies, further confirming the importance of addressing these behaviors in medical and healthcare education.

**Table 2:** Statistical tests for gender, year of the study, and various ethical behavior variables highlighting key areas for improvement in professional ethics education

Variable	Test type	Test statistic	Degree of freedom	Asymptotic sig. (2-tailed)	Standard error	Standardized test statistic
Age	One-sample Chi-square	1651.56	5	0	-	-
Gender	One-sample binomial	293	-	0	14.274	-7.986
Degree	One-sample binomial	674	-	0	14.274	18.635
Year of the study	One-sample Chi-square	128.95	4	0	-	-
Lack of maintaining medical dignity	One-sample Chi-square	627.724	3	0	-	-
Lack of respect for cultural differences	One-sample Chi-square	491.439	3	0	-	-
Lack of self-assessment	One-sample Chi-square	510.728	3	0	-	-
Lack of commitment to continuous learning	One-sample Chi-square	319.071	3	0	-	-
Lack of equity in serving patients	One-sample Chi-square	590.885	3	0	-	-
Lack of acceptance of health risks	One-sample Chi-square	398.463	3	0	-	-
Failure to comply with regulations	One-sample Chi-square	425.849	3	0	-	-
Lack of bearing discomfort in medical needs	One-sample Chi-square	513.26	3	0	-	-
Disregard for educational activities	One-sample Chi-square	364.058	3	0	-	-
Denial of errors and mistakes	One-sample Chi-square	445.285	3	0	-	-
Medical negligence	One-sample Chi-square	586.369	3	0	-	-
Dishonest behavior in the workplace	One-sample Chi-square	553.633	3	0	-	-
Lack of discipline in medical work	One-sample Chi-square	542.551	3	0	-	-
Lack of commitment when on-call	One-sample Chi-square	494.845	3	0	-	-
Preference of personal interests over patient	One-sample Chi-square	474.615	3	0	-	-
Suggesting unaffordable treatment options	One-sample Chi-square	485.618	3	0	-	-
Lack of commitment to patient privacy	One-sample Chi-square	522.301	3	0	-	-
Failure to maintain physician-patient privacy	One-sample Chi-square	516.745	3	0	-	-
Failure in teamwork duties	One-sample Chi-square	448.71	3	0	-	-
Failure to address patient feelings	One-sample Chi-square	516.931	3	0	-	-
Failure to maintain professional boundaries	One-sample Chi-square	495.915	3	0	-	-
Use of alcohol or drugs in the workplace	One-sample Chi-square	865.682	3	0	-	-
Addressing patients inappropriately	One-sample Chi-square	487.101	3	0	-	-
Lack of empathy with patients	One-sample Chi-square	527.935	3	0	-	-
Failure to report inappropriate colleague behavior	One-sample Chi-square	466.006	3	0	-	-
Failure to introduce oneself to patient and family	One-sample Chi-square	431.267	3	0	-	-
Performing procedures without supervision	One-sample Chi-square	489.191	3	0	-	-
Having personal conversations in the hospital	One-sample Chi-square	375.258	3	0	-	-
Eating or drinking in the hospital hallways	One-sample Chi-square	319.228	3	0	-	-
Familiarity with professional ethics	One-sample binomial	766	-	0	14.274	25.08
Passed a course in medical ethics	One-sample binomial	326	-	0	14.274	-5.675
Passed other professional ethics training	One-sample binomial	213	-	0	14.274	-13.591

On the other hand, some variables, such as "Familiarity with professional ethics," "Passed a course in medical ethics," and "Passed other professional ethics training," were analyzed using the one-sample binomial test, and these also show statistically significant results with p-values of 0. This indicates that familiarity and formal training in professional ethics are crucial components of the sample population's experiences. A particularly strong result is seen in the category of "Familiarity with professional ethics" with a standardized test statistic of 25.08, suggesting that the majority of respondents are familiar with the concept and application of professional ethics, which could point to a higher level of awareness and education regarding these principles.

Despite the overwhelming statistical significance of most of the variables, the results also highlight that some areas may require more attention. The "Degree" and "Year of study" variables, analyzed with one-sample binomial and one-sample Chi-square tests, respectively, also show significant results. These findings suggest that the year of the study and the type of degree (MBBS or BDS) may influence students' awareness and behavior related to professional ethics. The data reinforces the notion that ongoing education and training, especially concerning ethical conduct, are necessary across various stages of a medical or dental student's academic journey.

The results of the analysis in Table 3 show that several variables related to professional ethics exhibit varying levels of statistical significance (p-values), which help in understanding the differences in ethical behaviors across year groups and degree types. For example, "Lack of maintaining medical dignity in a relationship" shows a p-value of 0.0761, which is marginally above the conventional threshold for statistical significance (0.05), suggesting that there might be no significant difference in this behavior across different years of study or between MBBS and BDS students. However, while the p-value suggests no significant findings, the large frequency counts in the responses may indicate varying perceptions across different categories. The relatively high frequencies in the "Observed" and "Observed & participated" columns hint that while the overall p-value is not significant, the behavior is still notable in terms of student awareness and participation.

**Table 3:** Distribution of various unprofessional behaviors and their statistical significance across different categories of medical and dental students

Category	Degree	1st Year	2nd Year	3rd Year	4th Year	5th Year	Total	p-value	*x*^2^-value
Lack of maintaining medical dignity in a relationship	MBBS	25	66	47	44	16	158	0.0761	510.728
	BDS	41	129	140	125	56	73		
	Total	199	497	71	48	198	806		
Lack of respect for religious & cultural differences	MBBS	267	101	81	84	27	330	1.7325	122.305
	BDS	58	100	110	98	42	395		
	Total	325	395	390	498	198	815		
Lack of self-assessment & refusal to accept critiques	MBBS	205	83	58	56	22	448	2.4138	510.728
	BDS	44	109	131	118	72	51		
	Total	249	448	392	444	247	806		
Lack of commitment to continuous learning	MBBS	208	82	64	48	24	251	7.043	319.071
	BDS	43	99	113	95	42	379		
	Total	251	381	375	271	248	806		
Lack of equity & fairness in serving patients	MBBS	187	72	56	47	26	235	9.2635	590.885
	BDS	47	126	137	126	45	471		
	Total	234	475	469	372	230	806		
Lack of acceptance of health risks in front of patients	MBBS	279	107	85	73	27	332	7.8828	398.463
	BDS	53	90	103	93	46	355		
	Total	332	359	188	173	329	806		
Failure to comply with hospital regulations	MBBS	272	109	84	75	26	335	8.9628	425.849
	BDS	63	89	109	94	62	362		
	Total	335	365	393	302	332	806		
The lack of bearing difficulty in responding to patients	MBBS	264	110	80	70	30	328	6.2126	513.26
	BDS	62	93	114	103	44	396		
	Total	328	398	284	173	324	806		
Disregard educational activities	MBBS	146	61	36	41	14	178	3.9333	364.058
	BDS	34	116	122	101	42	417		
	Total	180	420	420	278	178	806		
Denial of errors, mistakes, & wrongdoing	MBBS	212	78	61	55	25	258	4.0033	445.285
	BDS	46	112	117	116	45	420		
	Total	258	423	468	172	255	806		
Medical negligence in duties	MBBS	220	85	63	59	23	272	1.1833	586.369
	BDS	52	113	132	120	46	453		
	Total	272	455	392	179	266	806		
Dishonest behavior in workplace	MBBS	245	85	73	75	29	299	5.4729	553.633
	BDS	58	115	124	109	42	424		
	Total	303	428	197	109	178	806		
Lack of observance of discipline	MBBS	193	79	54	54	19	235	5.2934	542.551
	BDS	46	115	130	121	46	456		
	Total	239	460	417	175	235	806		
Lack of commitment to be available and responsive when “on call”?	MBBS	286	310	50	28	345	674	4.2105	494.845
	BDS	64	64	9	4	141	141		
	Total	350	374	59	32	345	815		
Prefer their interests to the interests of the patient?	MBBS	289	303	59	23	674	674	0.9577	474.615
	BDS	62	63	10	6	141	141		
	Total	351	366	69	29	815	815		
Not suggesting treatment options to patients who cannot afford them?	MBBS	328	265	43	38	674	674	0.5866	485.618
	BDS	66	59	12	4	141	141		
	Total	394	324	55	42	815	815		
Lack of commitment to patient privacy?	MBBS	320	284	38	32	674	674	0.8894	522.301
	BDS	69	59	9	4	141	141		
	Total	389	343	47	36	815	815		
Failure to perform duties in teamwork?	MBBS	265	317	65	27	674	674	0.9495	448.71
	BDS	53	69	15	4	141	141		
	Total	318	386	80	31	815	815		
Play down feelings, needs, and wishes of the patient?	MBBS	310	294	43	27	674	674	0.7621	516.931
	BDS	72	55	7	7	141	141		
	Total	382	349	50	34	815	815		

On the other hand, other variables show more substantial differences across groups. "Lack of respect for religious & cultural differences" with a p-value of 1.7325, "Lack of self-assessment & refusal to accept critiques" (p-value = 2.4138), and "Lack of commitment to continuous learning" (p-value = 7.0430) indicate significant results with p-values well below 0.05. These findings suggest that there is a notable difference in how medical (MBBS) and dental (BDS) students view these issues, and these differences may be associated with the specific education or training students undergo in their respective fields. In these categories, MBBS students show higher frequencies of negative behaviors, while BDS students generally report a more positive view of these ethics-related issues, which could be indicative of a different academic focus or exposure to professional conduct training.

For other variables such as "Failure to perform duties in teamwork," "Not suggesting treatment options to patients who cannot afford them," and "Failure to maintain professional boundaries," the p-values (ranging from 0.5866 to 0.9577) suggest that no significant differences exist between the groups. These results imply that there is a broad consensus across both degree programs and year groups, with little variation in how students perceive these behaviors. Despite this, the frequency of responses in these categories still reveals the importance of addressing such issues during medical and dental education. These findings can inform targeted interventions to improve specific areas of professional behavior, particularly where no significant differences in student perceptions were found.

## Discussion

The findings from our study on the awareness, training, and observation of professional ethics among medical (MBBS) and dental (BDS) students reveal significant insights. Our finding shows that the majority of participants are young adults aged 20-22 years and that there is a noticeable gender imbalance with a higher proportion of female participants compared to males. Williams et al. [11] indicated that younger medical students and female participants tend to be more involved in ethics courses and highlighted that female healthcare professionals often report higher ethical sensitivity and empathy compared to their male counterparts.

The significant skew toward MBBS students (82.7%) versus BDS students (17.3%) may reflect broader trends in medical education where MBBS programs generally include more structured ethical training compared to dental programs. Current results indicating that the second and third years have higher engagement in self-directed learning and awareness of professional ethics are supported by Bazrafcan et al. [12] suggesting that middle years of study are critical for the inculcation of professionalism. This period is often when students begin to face real-world ethical dilemmas.

A high percentage of participants (94%) report familiarity with professional ethics, yet a significant portion (60%) have not taken formal medical ethics courses. This aligns with prior studies that suggest that medical curricula often emphasize clinical skills over structured ethics education [13]. The disparity between awareness and formal training in current educational strategies supports the call for integrating ethics more comprehensively into medical and dental training.

Our study reveals that 59.3% of participants engaged in self-directed study on professionalism. This is consistent with findings in another study that highlight an increasing trend among students to independently explore areas related to medical ethics, especially in the absence of formal curriculum coverage [14]. Lee et al. [15] also indicate that students who pursue self-directed learning profess better ethical decision-making and patient-centered care.

A number of participants reported observing unprofessional behaviors, such as failure to maintain medical dignity (61%) and disregard for religious and cultural differences (48.5%). This corresponds with a study that identifies these issues as prevalent in clinical settings, particularly in environments where students are not explicitly taught to address such situations [16]. The findings of unprofessional behaviors being observed but less frequently participated in suggest a passive-active engagement. Similar trends have been reported where students, especially in their early years, feel reluctant to challenge unethical behaviors due to hierarchical dynamics in healthcare settings [17,18].

Our analysis uses the Mann-Whitney gender differences in areas like familiarity with ethics, self-directed study, and formal training. This supports previous studies indicating that gender can influence perceptions and engagement with ethics education, with female students often showing a greater interest in and commitment to ethical issues [18,19]. The results suggest that male and female students experience the medical environment differently, potentially due to varying socialization patterns or within medical schools.

The statistically significant differences between MBBS and BDS like maintaining medical dignity, respect for cultural differences, and continuous learning reflect different training emphases in these programs. Literature often highlights that MBBS programs are more rigorous in ethics training compared to dental programs [20]. Interestingly, BDS students reported a higher frequency of observed ethical violations but showed a more positive view overall, which may indicate differences in clinical exposure by dental versus medical students.

The Chi-square analysis highlights widespread recognition of issues like "failure to maintain professional boundaries" or "empathy," which are commonly reported in healthcare literature as critical concerns impacting patient care. However, the lack of significant differences across certain variables (e.g., failure to introduce oneself) suggests that despite different training backgrounds, students across disciplines perceive certain unprofessional behaviors similarly. This aligns with findings that emphasize the universal nature of professionalism across healthcare fields [21].

Strength and limitations

A key strength of this study lies in its large and diverse sample, which included medical students across various academic years and institutions. This diversity allowed for a broad understanding of perceptions and experiences related to professionalism and ethics, capturing the nuances of different training stages. The use of a structured and validated questionnaire provided reliable insights into factors such as gender differences, self-directed learning, and real-world observations of ethical practices, making the findings particularly relevant for enhancing medical and dental curricula.

However, certain limitations must be acknowledged. The reliance on self-reported data may introduce biases, such as social desirability bias, where participants may provide responses that align with perceived expectations rather than their true experiences. Additionally, the cross-sectional design offers a snapshot of students' perceptions and experiences but cannot account for changes or developments over time. Another limitation is the sample's restriction to specific institutions in Pakistan, which may limit the generalizability of the findings to other regions or educational systems.

To address these limitations in future research, longitudinal studies that follow students throughout their academic and clinical training are recommended. Such studies could provide deeper insights into the evolution of ethical perceptions and behaviors over time. Expanding the sample to include diverse medical schools and regions could further enhance the generalizability of findings and offer a more comprehensive understanding of professionalism in medical education.

## Conclusions

The study reveals that while the majority of participants, primarily young adult females enrolled in MBBS programs, demonstrate substantial familiarity with professional ethics, significant gaps remain in formal ethics education and practical training. Despite a general awareness of ethical conduct, many students lack structured exposure to medical ethics, with gender differences evident as females are more likely to engage in self-directed study on ethics. Clinical experiences influenced students' understanding, though the prevalence of unprofessional behaviors, such as neglecting medical dignity and cultural sensitivity, indicates that real-world practice often falls short of ethical ideals.

These findings underscore the critical need for integrating ethics education into medical and dental curricula through a more structured and practical approach. Strategies could include embedding ethics modules across all academic years, incorporating case-based discussions and role-playing scenarios, and aligning ethical training with clinical rotations to provide real-time context for ethical decision-making. The institutional policies should encourage open discussions on professionalism and ethical dilemmas, supported by faculty training to model and reinforce ethical behaviors. Future research could focus on evaluating the effectiveness of these interventions in strengthening ethical practices among healthcare professionals.

## Appendices

**Table 4:** Participant response collection form MBBS: Bachelor of Medicine and Bachelor of Surgery; BDS: Bachelor of Dental Surgery

Field/question	Participant input
Timestamp	
Email address	
Are you voluntarily giving your consent as a participant in this research?	(Select one: yes/no)
Referral code provided by the volunteer	
Age	
Gender	(Select one: male/female/other)
Degree	(e.g., MBBS, BDS)
Year of study	(e.g., 1st year, 2nd year)
Are you familiar with the meaning and application of professional ethics?	(Select one: yes/no)
Have you passed a course in medical ethics before?	(Select one: yes/no)
Have you passed another training course, other than a medical ethics course, about professional ethics?	(Select one: yes/no)
Do you have any self-directed study in the context of professionalism?	(Select one: yes/no)
Lack of maintaining medical dignity in their relationship (talking, dressing) with patients/colleagues?	(Select one: observed only/participated only/both observed & participated/neither)
Lack of respect for people's (patients, colleagues) religious and cultural differences?	(Select one: observed only/participated only/both observed & participated/neither)
Lack of self-assessment and refusal to accept and apply constructive critiques?	(Select one: observed only/participated only/both observed & participated/neither)
Lack of commitment to continuous learning?	(Select one: observed only/participated only/both observed & participated/neither)
Lack of equity and fairness in serving patients?	(Select one: observed only/participated only/both observed & participated/neither)
Lack of acceptance of probable health risks in front of the patient?	(Select one: observed only/participated only/both observed & participated/neither)
Failure to comply with hospital regulations and policy?	(Select one: observed only/participated only/both observed & participated/neither)
Lack of bearing difficulty and discomfort in responding to the medical needs of patients?	(Select one: observed only/participated only/both observed & participated/neither)
Disregard for educational activities (e.g., arriving late to rounds for nonclinical reasons, skipping required lectures/seminars)?	(Select one: observed only/participated only/both observed & participated/neither)
Denial of any errors, mistakes, and wrongdoing?	(Select one: observed only/participated only/both observed & participated/neither)
Medical negligence in duties in the hospital setting?	(Select one: observed only/participated only/both observed & participated/neither)
Dishonest behavior in the workplace?	(Select one: observed only/participated only/both observed & participated/neither)
Lack of observance of discipline in medical work?	(Select one: observed only/participated only/both observed & participated/neither)
Lack of commitment to be available and responsive when “on call”?	(Select one: observed only/participated only/both observed & participated/neither)
Preferring personal interests to the interests of the patient?	(Select one: observed only/participated only/both observed & participated/neither)
Not suggesting treatment options to patients who cannot afford them?	(Select one: observed only/participated only/both observed & participated/neither)
Lack of commitment to patient privacy?	(Select one: observed only/participated only/both observed & participated/neither)
Lack of commitment to the privacy of the patient-physician relationship?	(Select one: observed only/participated only/both observed & participated/neither)
Failure to perform duties in teamwork?	(Select one: observed only/participated only/both observed & participated/neither)
Playing down feelings, needs, and wishes of the patient?	(Select one: observed only/participated only/both observed & participated/neither)
Failure to maintain a professional boundary in relation to patients or colleagues?	(Select one: observed only/participated only/both observed & participated/neither)
Use of alcohol or drugs in the workplace?	(Select one: observed only/participated only/both observed & participated/neither)
Addressing a patient inappropriately?	(Select one: observed only/participated only/both observed & participated/neither)
Lack of empathy and compassion with patients?	(Select one: observed only/participated only/both observed & participated/neither)
Failure to report the risky and/or inappropriate behavior of a colleague (after approaching the individual)?	(Select one: observed only/participated only/both observed & participated/neither)
Failure to introduce yourself and nurses to the patient and their family?	(Select one: observed only/participated only/both observed & participated/neither)
Performing procedures without sufficient skills (without supervision)?	(Select one: observed only/participated only/both observed & participated/neither)
Having personal conversations or making fun of students, other physicians, peers, or staff in corridors of the hospital?	(Select one: observed only/participated only/both observed & participated/neither)
Eating or drinking in the hallway of the hospital?	(Select one: observed only/participated only/both observed & participated/neither)
